# Methadone Induction in Primary Care for Opioid Dependence: A Pragmatic Randomized Trial (ANRS Methaville)

**DOI:** 10.1371/journal.pone.0112328

**Published:** 2014-11-13

**Authors:** Patrizia Maria Carrieri, Laurent Michel, Caroline Lions, Julien Cohen, Muriel Vray, Marion Mora, Fabienne Marcellin, Bruno Spire, Alain Morel, Perrine Roux, the Methaville Study Group

**Affiliations:** 1 INSERM UMR912 (SESSTIM), Marseille, France; 2 Aix Marseille Université, UMR_S912, Marseille, France; 3 ORS PACA, Observatoire Régional de la Santé Provence Alpes Côte d'Azur, Marseille, France; 4 INSERM, Research Unit 669, Paris, France; 5 Univ Paris-Sud and Univ Paris Descartes, UMR-S0669, Paris, France; 6 Centre Pierre Nicole, Paris, France; 7 Unité de Recherche et d'Expertise en Epidémiologie des Maladies Emergentes, Institut Pasteur, Paris, France; 8 Institut National de la Santé et de la Recherche Médicale (INSERM), Paris, France; 9 Oppelia, Paris, France; California Pacific Medical Center Research Institute, United States of America

## Abstract

**Objective:**

Methadone coverage is poor in many countries due in part to methadone induction being possible only in specialized care (SC). This multicenter pragmatic trial compared the effectiveness of methadone treatment between two induction models: primary care (PC) and SC.

**Methods:**

In this study, registered at ClinicalTrials.Gov (NCT00657397), opioid-dependent individuals not on methadone treatment for at least one month or receiving buprenorphine but needing to switch were randomly assigned to start methadone in PC (N = 155) or in SC (N = 66) in 10 sites in France. Visits were scheduled at months M0, M3, M6 and M12. The primary outcome was self-reported abstinence from street-opioids at 12 months (M12) (with an underlying 15% non-inferiority hypothesis for PC). Secondary outcomes were abstinence during follow-up, engagement in treatment (i.e. completing the induction period), retention and satisfaction with the explanations provided by the physician. Primary analysis used intention to treat (ITT). Mixed models and the log-rank test were used to assess the arm effect (PC vs. SC) on the course of abstinence and retention, respectively.

**Results:**

In the ITT analysis (n = 155 in PC, 66 in SC), which compared the proportions of street-opioid abstinent participants, 85/155 (55%) and 22/66 (33%) of the participants were classified as street-opioid abstinent at M12 in PC and SC, respectively. This ITT analysis showed the non-inferiority of PC (21.5 [7.7; 35.3]). Engagement in treatment and satisfaction with the explanations provided by the physician were significantly higher in PC than SC. Retention in methadone and abstinence during follow-up were comparable in both arms (p = 0.47, p = 0.39, respectively).

**Conclusions:**

Under appropriate conditions, methadone induction in primary care is feasible and acceptable to both physicians and patients. It is as effective as induction in specialized care in reducing street-opioid use and ensuring engagement and retention in treatment for opioid dependence.

**Trial registration:**

Number Eudract 2008-001338-28; ClinicalTrials.gov: NCT00657397; International Standard Randomized Controlled Trial Number Register ISRCTN31125511

## Introduction

Methadone is included in the WHO list of essential medicines thanks to its effectiveness in treating opioid dependence, preventing HIV [1] and improving adherence to antiretroviral treatment in HIV-infected individuals [2]. Despite this, access to methadone remains limited because of the risk of overdose during induction, especially in countries where the need for methadone is even greater.

While access to buprenorphine in primary care has been possible since 1996 thanks to its safety profile [3], [4], methadone induction in France, as in most countries, is currently possible only in specialized centers caring for substance dependence (located in *ad hoc* sites or in hospitals) (hereafter specialized care or SC). In France these centers can refer patients to PC only after the end of methadone induction, i.e. when methadone doses are stabilized (after at least 14 days).

The specific model of care for regulating methadone induction can greatly influence its safety as the risk of overdose during the induction phase remains a major concern. Internationally, the regulations governing the extent to which methadone induction (i.e. until dosage stabilization) is authorized in primary care (PC) differ considerably. For example, methadone induction in PC is legal in the UK, in Switzerland and in Canada under different models of care. In contrast, France, the United States but also other countries have no such system currently in place.

This means that in the many geographic areas underserved by SC, opioid-dependent individuals seeking treatment have no access to methadone. To tackle this situation, one of the objectives of the French public health authorities' national strategic plan for prevention and care of Hepatitis was to consider using primary care as an entry point for methadone treatment, based on the results of a pragmatic trial. The trial, entitled Methaville, was designed both to evaluate the feasibility of methadone induction in PC and to compare outcomes in participants randomized into PC induction with those randomized into SC induction. Being a pragmatic trial, the objectives were to verify the feasibility and acceptability of the PC induction model to physicians and patients, and also to show that the main patient outcome (street-opioid abstinence after one year of treatment) and secondary outcomes (abstinence during follow-up, engagement in treatment, retention in treatment and satisfaction with the explanations provided by the physician) were all comparable between both induction arms.

## Methods

The protocol for this trial and supporting CONSORT checklist are available as Checklist S1 and English protocol S1.

### Ethics

The Methaville ANRS trial is registered with the French Agency of Pharmaceutical Products (AFSSAPS) under the number 2008-A0277-48, the European Union Drug Regulating Authorities Clinical Trials: Number Eudract 2008-001338-28, the ClinicalTrials.gov Identifier: NCT00657397 and the International Standard Randomized Controlled Trial Number Register ISRCTN31125511. The study protocol was approved by the Ethics Committee of Persons Protection in Paris, France. All individuals provided written, informed consent before participating in the study.

### Physicians and Participants

In this multicenter, pragmatic, randomized trial, for each study site we selected an SC physician and nearby PC physicians who already had field experience in care for opioid dependence and/or training in care for drug dependence, and who were willing to participate. Only PC physicians with patients potentially eligible for enrollment in the study were selected because if methadone induction in PC is officially adopted in France in the future, only PC physicians meeting the above criteria will be targeted by authorities as methadone prescribers. Ten sites in four geographic areas (North, North-Eastern, South-Western and South-Eastern France) were included, with each site having at least one SC and one PC physician. These four geographical areas were chosen to better target the different types of populations who would benefit from access to methadone in primary care, as each geographical area is characterized by different drug markets and drug user practices/needs. Physicians (in PC and SC) enrolled opioid-dependent individuals who were randomized to start methadone either in PC or in SC.

Inclusion criteria were chosen to target a population representative of drug users needing/seeking methadone treatment in France as follows: aged over 18 years old, seeking care for opioid dependence and not prescribed methadone for at least one month or receiving buprenorphine but needing to switch to methadone for medical reasons (side effects, treatment misuse, etc.). Non-inclusion criteria were similar to those in other studies involving methadone prescription in PC [5], [6] as follows: could not be reached by phone for an interview and screening positive for opioids/benzodiazepines/alcohol triple co-dependence, as assessed by the MINI [7] (as this condition exposed participants to a high risk of overdose [8], [9]) and finally, for women, being pregnant.

### Study design

The first visit took place when a patient seeking care for opioid dependence or needing to switch treatment from buprenorphine to methadone went to see a PC or SC physician participating in the trial. After providing consent to participate the patient was randomized into the PC or SC arm by this physician. To make patient randomization feasible, each site had one SC with at least one PC physician in the nearby vicinity. All PC and SC physicians involved underwent a one-day training course both to standardize methadone induction practices according to trial guidelines and to acquaint them with trial procedures [10]. Trial guidelines stipulated that the starting dose should be on average 30 mg and not exceed 40 mg, with 10 mg increases every 2–4 days thereafter until dose stabilization. This is comparable to methadone induction protocols used in other trials [5], [11].

The “intervention” provided by trained physicians consisted in at least fourteen-day supervised methadone induction either in PC or in SC according to trial guidelines (Table 1). Thereafter, supervision was required only for patients who were deemed to be at risk of overdose. One main difference between both arms in the model of care was that delays in initiating treatment were more common in SC (see Table 1).

**Table 1:** Features of the PC and SC model of care for methadone treatment (ANRS Methaville trial).

Methaville model for Primary Care (PC)	Current Methadone model for specialized care (SC)
• During induction, methadone intake is delivered and supervised daily at the pharmacy (with take home doses only for the weekend).Supervision is compulsory during induction.	• During induction, methadone is delivered daily at the center by the physician, the pharmacist or the nurse or is delivered at the pharmacy (with take home doses only for the weekend).Supervision is compulsory during induction.
• Psychosocial and health status assessment is not a necessary condition to start methadone – referral to specialized center if needed.	• It is recommended that Methadone induction is started after initial visits/interviews carried out by different members of health staff:
	a) A social counselor and/or a psychologist to obtain a psychosocial assessment of the patient;
	b) A physician or a nurse to obtain an assessment of the general health of the patient;
	c) An assessment of his/her social rights (health insurance, accommodation, resources, and previous access to care for drug dependence).
• Referral to psychosocial counseling in SC during methadone treatment if needed.	• Psychosocial counseling provided during methadone treatment.
• Methadone prescription possible the same day as the first medical visit.	• Time before methadone prescription may be delayed by some days after the first medical visit, depending on patient's conditions (withdrawal syndromes, pregnancy, etc.).
• Doses are prescribed according to Methaville guidelines.	• Doses are prescribed according to Methaville guidelines.
• Doses are reassessed at every medical visit (i.e. every 2–3 days) during induction.	• Doses are reassessed at every medical visit (i.e. every 2–3 days) during induction.
• Urine analyses at first dose prescription and monitoring once/twice a week during induction.	• Urine analyses at first dose prescription and monitoring once/twice a week during induction.

Participants were also followed up over one year through medical visits and phone interviews at months 0 (M0, enrolment), at 3, 6 and 12 months (M3, M6 and M12, respectively).

To emulate current care practices in France, patients could choose to change arm after the induction phase, i.e. SC physicians could refer their patient to a PC colleague who would prescribe methadone and vice versa. Accordingly, arm changes (PC to SC or SC to PC) were not considered as deviations from the protocol. Further details and the pre-trial phase of the protocol are described elsewhere [10].

### Data sources and outcomes

The following 5 sources provided study data: a pre-enrollment medical questionnaire, a medical questionnaire at each scheduled visit (enrolment (M0), months 3, 6 and 12, respectively M3, M6 and M12), a short self-administered questionnaire (completed during scheduled visits), a Computer Assisted Telephone Interview (CATI) (conducted just after each scheduled visit) lasting no more than 30 minutes, and when available, urine rapid tests [10].

Abstinence from street-opioids at M12 was chosen as the primary outcome. Abstinence during the course of treatment, engagement in treatment, retention in methadone maintenance treatment and patient satisfaction with the explanations provided by the physician were secondary outcomes. The primary outcome, abstinence from street opioids during the previous month, was measured using a validated question [12], administered during phone interviews by trained non-judgmental staff. This question was also answered in the medical interviews at enrolment, M3, M6 and M12.

Methadone dose was defined as the number of mg/day of methadone prescribed by the physician for each patient at each medical visit.

Engagement in treatment was computed in those randomized to each specific arm as the proportion of patients who actually started methadone and remained in the trial until the stabilization of dosages (i.e. until the end of induction). Follow-up participation rates, i.e. participants who continued participating in study interviews until M12, were computed for each arm.

Retention in methadone maintenance treatment was defined, only for patients who actually started methadone treatment, as the time between the first day of methadone induction and the last known date that the patient was still receiving treatment. For individuals still on methadone at M12, retention was set at 12 months.

A 5-point Likert scale measured patient satisfaction with the explanations provided by his/her prescribing physician during CATI at M6 only. This outcome was then dichotomized (very satisfied vs. other).

There were three main reasons to justify the choice to use patient self-reported street-opioid use as the primary outcome. The first reason was to avoid introducing measures which would not reflect routine practices in the field of care for drug dependence in France (for example neither urine tests nor hair analysis are routinely performed). These alternative measures would have been negatively affected by several missing values. Furthermore, any cases with complete values would most likely have been biased, as urine testing is currently performed at the discretion of the physician if he/she is doubtful about patients' drug use. The second one was to place the patient at the center of the study and consider his/her self-reported experience with treatment (satisfaction with the explanations provided by the physician) as a major outcome. This choice reflects the work in other trials involving drug users and in other fields of medicine where patient's reported outcomes are considered as main outcome measures [13], [14]. Third, patient self-reports are valid as PC physicians rely on them in clinical practice [15].

We chose the endpoint at M12 for the trial phase on the basis that any comparison between arms would not be significant enough immediately after the induction phase or indeed at M3, and that any possible benefit could be confirmed after one year of treatment.

The outcomes used in this pragmatic trial were those typically used in trials assessing the efficacy of a treatment for opioid dependence [5], [6]. The only difference here is that they were used to assess methadone effectiveness (and not efficacy) by induction arm.

### Safety issues

To ensure safety during induction, we implemented a wide range of strategies minimizing overdose risk in the trial's model of care, including a one-day training session for PC physicians, strict supervision of participants when they took their doses at the pharmacy during treatment initiation, and fostering strong collaboration between all the health professionals involved in the study, including pharmacists (shared-care model) [16]. The non-inclusion of patients with triple co-dependence (alcohol-opioid-benzodiazepine) [8], [9] does not constitute a restrictive criterion and reflects standard practice for safety concerns. Methadone initiation in these patients is possible only after benzodiazepine detoxification.

Pharmacists and physicians involved in the trial had to signal overdoses, signs of intoxication and lost-to-follow-up patients to the center of methodology and management (CMM) (ORS PACA- INSERM-IRD UMR912). The latter was required to notify any severe adverse event, such as an overdose, to the French National Agency for Research on Aids and Viral Hepatitis (ANRS) which in turn notified the French Agency of Pharmaceutical Products (ANSM).

A list of 50 health-related symptoms included in the questionnaire helped document self-reported symptoms during follow-up.

### Statistical considerations

#### Sample size

Although retention in treatment is the most important outcome in patients receiving treatment for opioid dependence [17] and even more important in the context of decision-making by public health authorities, this trial was funded for HCV prevention purposes with opioid abstinence being considered the most pertinent primary outcome. It was therefore designed with an underlying hypothesis of non-inferiority in terms of the proportion of PC-inducted patients who were abstinent from street-opioids at 12 months (M12) of methadone treatment with respect to their SC counterparts.

The study hypothesis was that after one year of treatment, the proportion of patients abstinent from street-opioids would be 70% [18], [19] for SC. Selecting an inferiority margin of 15 for patients starting methadone in PC entailed recruiting a minimum of 150 patients in order to show the non-inferiority, if any, of the PC arm for the primary outcome.

The choice of this margin reflected our willingness to accept decreased effectiveness in PC in return for increased attractiveness and engagement in PC treatment.

#### Randomization and masking

Randomization of patients was performed centrally by the study's methodology and data management center (ORS PACA- INSERM-IRD UMR912), via a secured intranet site, by simple random sampling with no block control on randomization rate. Information about patient randomization was confidentially stored and hidden from the study research team - except statisticians and the data manager - until the end of the last M12 interview, in December 2011. A randomization ratio 2∶1 (PC: SC) was chosen to deliberately over-represent patients followed-up in PC to increase the probability of detecting possible intoxications in PC during induction.

#### Statistical analysis

Medians and interquartile ranges (IQR) and proportions were used to describe the distributions of continuous and categorical variables, respectively. Distributions of variables among the two groups were compared using Mann-Whitney U test for continuous variables, Chi-Squared or Fisher exact test for categorical variables.

**Figure 1:**
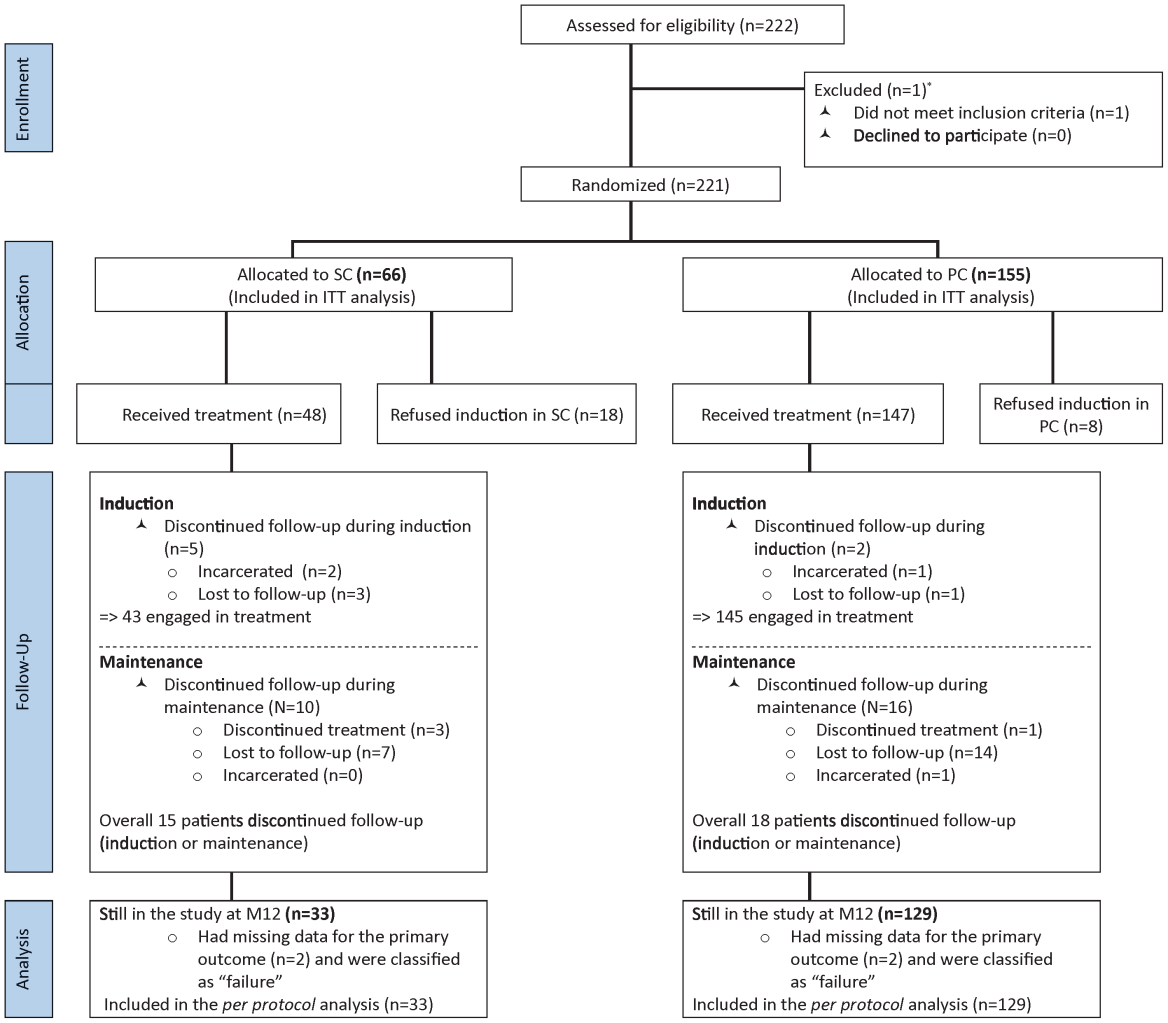
Flow chart of ANRS Methaville trial.

The primary analysis used intention-to-treat (ITT) (n = 221) and the primary outcome was measured using a validated question [12] about opioid use during the previous month collected by CATI or medical interview (when CATI data was incomplete). The difference between both arms in the proportion of patients reporting abstinence from street-opioids during the previous month at M12 and the related 95% confidence interval (95% CI) were computed. In this ITT analysis all patients who discontinued follow-up before M12 for any reason (i.e. refused to start methadone after randomization or discontinued follow-up for any reason including treatment interruption, lost to follow-up, incarceration etc.) were classified as “failure” i.e. street-opioid users (see Fig. 1).

A “*per protocol*” analysis was also conducted only on individuals still followed-up at month 12 (n = 162).

Participants still followed up at M12 but with missing values for OTI (Opiate Treatment Index) at M12 were also classified as street-opioid users in both the ITT and *per protocol* analysis. The ITT classification for those who discontinued follow-up for any reason and for those with missing data is particularly pertinent in this population of opioid-dependent individuals because discontinuation is generally associated with relapse into street-opioid use.

Logistic mixed models assessed the effects of time on methadone and of each arm on the course of abstinence from street-opioids, and took into account whether patients were switching from buprenorphine to methadone at enrolment. Mixed models are currently considered the most appropriate methods to use in clinical trials with missing outcomes, as they meet intention-to-treat criteria [20]. A log-rank test was used to compare Kaplan–Meier curves for retention in treatment among patients who were engaged in treatment, i.e. completed the induction phase (n = 188). SAS (v.9.2) and STATA (v.12) statistical software packages were used for the statistical analyses.

## Results

### Sites and physicians included in the study

Of 12 sites contacted in France, two refused to participate for organizational reasons. The 10 participating sites each included a SC and between 1 to 3 PC physicians in the nearby vicinity. Among the 57 physicians (SC & PC) who agreed to participate, 32 (56%) enrolled at least one patient who met the inclusion criteria. These 32 physicians were significantly different from the other 25 in that they were significantly older (p = 0.02) and had more years of medical experience (p = 0.002).

### Baseline data

The patients included were mainly men (84%), median [IQR] age was 32 [27–38] years and 51% were switching from buprenorphine. Table 2 reports the main patient characteristics for each induction arm.

**Table 2:** Patient characteristics by induction arm (SC and PC) at baseline (ANRS Methaville trial).

	SC (n = 48) % or median [IQR]	PC (n = 147) (%) or median [IQR]	Total % or median [IQR]
Gender	21	14	16
Male	79	86	84
Female	21	14	16
Age - *years*	30 [27–39]	32 [27–38]	32 [27–38]
Employment	44	53	51
High school certificate	43	32	35
Living in a couple	33	31	32
Children	33	39	38
Home owner or renter	56	64	62
Living area			
Urban	59	52	54
Suburban	13	26	23
Rural	28	22	23
Switching from buprenorphine	52	51	51
Age at first drug use - *years* (n = 176)	18 [17]–[21]	18 [17]–[22]	18 [17]–[21]
Age at first regular drug use - *years* (n = 160)	20 [18]–[24]	20 [18]–[25]	20 [18]–[24]
History of drug injection (n = 175)	55	47	49
Age at first drug injection (n = 86) – years	22 [20]–[25]	21 [19]–[26]	22 [19]–[26]
Drug injection (n = 162)*	21	14	15
Drug snorting (n = 162)*	74	61	64
Use of street opioids (n = 187)*	79	69	72
Cocaine use (n = 162)*	26	27	27
Use of psychotropic drugs (n = 162)*	13	23	20
Daily cannabis use (n = 176)*	20	17	18
Hazardous alcohol consumption (n = 172)**	33	32	33
Depressive symptoms (n = 170)***	32	41	39
History of suicide attempts (n = 157)	10	18	17
History of drug overdose (n = 188)	12	12	12
HIV+ (n = 152)****	3	2	2
HCV+ (n = 140) ****	18	19	19

*during the previous 4 weeks.

**AUDIT score ≥7 for males and ≥6 for females.

***CES-D score>17 for males and>23 for females.

****among those who had already done a test.

### Patient disposition

From January 2009 to December 2010, the 32 physicians in the 10 trial sites enrolled 221 eligible individuals who were to be followed up for 12 months. The flow of participants through each stage is reported in Figure 1. All participants approached agreed to participate in the trial before randomization. One pregnant woman was excluded.

Among the 221 eligible patients, 66 and 155 were randomly assigned to start methadone in SC and in PC, respectively. However, 18 (27%) and 8 (5%) subsequently refused to be inducted in SC and in PC (p<0.001), respectively. These 26 patients were classified as “failure” in the ITT analysis.

**Figure 2:**
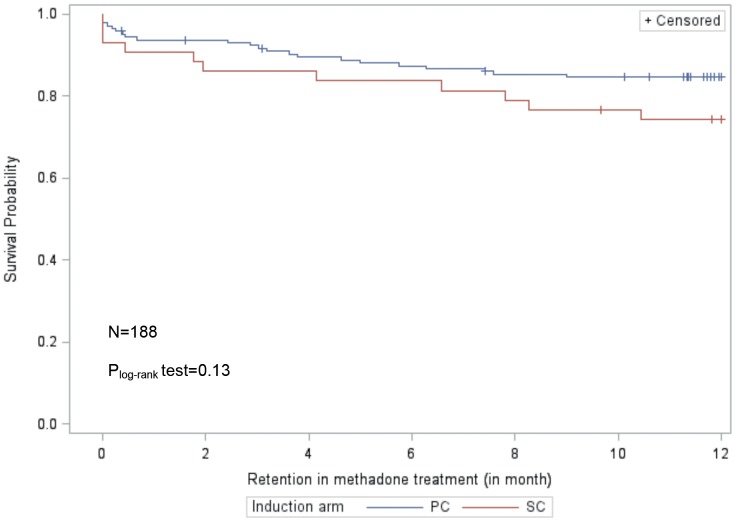
Retention in methadone maintenance treatment in patients (who completed the induction phase) in primary care (PC) versus those who started in specialized care (SC).

Of the 185 who started treatment, 5 SC and 2 PC patients dropped out (discontinued study visits) during induction (p = 0.026) (Figure 2) and were also classified as “failure” in the ITT analysis. Ten SC and 16 PC patients discontinued follow-up after induction (p = 0.13). They too were classified as “failure” in the ITT analysis.

Overall, 15 (31%) of the 48 SC-inducted patients and 18 (12%) of the 147 PC-inducted patients (p = 0.0023) discontinued follow-up. Reasons for discontinuation are reported in Figure 1.

Finally, 33 SC-inducted and 129 PC-inducted patients were present at M12 for the *per protocol* analysis. Data on the primary outcome was missing for four of these individuals (2 in SC and 2 in PC) who were therefore considered opioid users (i.e. “failure”) in the ITT and *per protocol* analyses.

Participants who were switching from buprenorphine were older, more likely male, and, most importantly, had a higher abstinence rate from street-opioids (38% vs. 12.5%) and cocaine (78.5% vs. 68%) at baseline, than those who were out of opioid maintenance treatment. However, as these differences were similar in both induction arms their compatibility in terms of the primary outcome remains unaffected.

Among the 48 SC patients, 18 (14 before M3 and 4 others before M6) changed to PC after induction, while one PC patient changed to SC. Nine patients were re-inducted during the study period after methadone interruption, two of these being re-inducted twice.

Sixty percent of the participants were prescribed methadone doses of between 60 and 80 mg between 11 and 17 days after initiation.

HIV and HCV self-reported prevalence rates were 2% and 19%, respectively.

The end of the study was set for the 1^st^ January 2012 to allow one full year of follow-up for patients enrolled in December 2010.

### Outcomes

Primary outcome was street opioid abstinence at 12 months and secondary outcomes: abstinence during follow-up, engagement in treatment, retention in treatment and satisfaction with the explanations provided by the physician.

The rates of participants included in the study and still in treatment at M12 were 129/155 = 83% for PC, 33/66 = 50% for SC and 162/221 = 73% for total sample.

The ITT and *per protocol* analyses reporting the difference in the proportions of street-opioid abstinent participants are described in Table 3. In the former (n = 155 in PC, 66 in SC), 85/155 (55%) and 22/66 (33%) were classified as street-opioid abstinent at M12 in PC and SC, respectively. The ITT analysis demonstrated the non-inferiority of the PC arm: the difference between the percentages of patients abstinent from street-opioids between both arms at M12 and the 95% CI was 21.5 [7.7; 35.3] in favor of PC. Although a *per protocol* analysis is not generally considered suitable in a pragmatic trial [15], it was computed for the sake of performing a comprehensive analysis. As expected, it provided inconclusive results, as the difference between the proportions and the non-inferiority margins was −0.8 [−18.8; 17.3], including the inferiority margin of −15.

**Table 3:** ITT and *per protocol* analysis for the difference in the percentage of street-opioid abstinent patients by induction arm and its 95% confidence interval.

	Specialized care	Primary care	% [95% CI] of the difference
**ITT analysis**			
Number of street-opioid abstinent patients at M12	22	85	
Number of patients included	66	155	
Street-opioid abstinent patients	33%	54%	21.5 [7.7; 35.3]
***Per protocol*** ** analysis**			
Number of street-opioid abstinent patients at M12	22	85	
Number of patients at M12	33	129	
Street-opioid abstinent patients at M12	67%	66%	−0.8 [−18.8; 17.3]

Mixed models found no significant arm effect (p = 0.39) on abstinence during follow-up, even when adjusting for the switch from buprenorphine treatment. Abstinence from street-opioids significantly increased between enrolment and M3 and remained stable thereafter (Table 4). These results remained valid even after adjusting for a possible heterogeneity effect among sites (i.e. study sites with more than one PC physician versus sites with only one) which was tested as a random effect factor in the mixed models but which was not found to be statistically significant.

**Table 4:** Odds ratio from the adjusted mixed model for abstinence from opioids use during the treatment (n = 615 visits and 188 patients).

	OR (IC95%)
**Time since methadone treatment initiation**	
M3 vs. M0	19.62 (8.69–44.33)
M6 vs. M0	16.73 (7.73–36.19)
M12 vs. M0	19.42 (8.98–41.98)
**Arm induction**	
PC vs. SC	1.58 (0.57–4.37)
**switching from buprenorphine**	
Yes vs. No	1.99 (0.85–4.67)

Engagement in treatment among those who were randomized only concerned patients who completed the methadone induction phase, i.e. 65% (43/66*100) for SC and 94% (145/155*100) for PC, p<0.001 (Fig. 1)).

When we computed “retention in treatment” we only focused on patients who had in fact started methadone treatment (i.e. 48 in SC and 147 in PC). At M12, thirty-three (33/48*100 = 69%) patients inducted in SC and 129 (129/147*100 = 88%) inducted in PC were still in treatment.

The Kaplan-Meier curves in Fig. 2 show that retention in methadone maintenance treatment was comparable between both induction arms.

Interestingly, PC-inducted patients reported high satisfaction with the explanations provided by their physician more often than their SC-inducted counterparts (p = 0.01).

### Prescribed methadone dose during the study

Regarding the median dose of prescribed methadone, there were no significant differences between both arms. The median [IQR] dose of methadone at M12 was 60 [45–90] mg in primary care and 67.5 [50–82.5] mg in specialized care.

### Adverse events and self-reported symptoms

No overdose was observed during the induction phase but one patient with a history of suicide attempts did intentionally overdose during methadone maintenance. Apart from this case, no other severe adverse events were reported during the trial.

The following symptoms were reported by more than 20% of patients at month 3: fatigue/energy loss (49%), difficulty sleeping (48%), constipation (40%), shortness of breath (33%), muscle pain (32%), tingling (32%), poor appetite (31%), wheezing (31%), loss of sexual desire (31%), stomach pain (28%), headaches (28%), joint pain (23%), weight loss (20%) and blackouts (20%).

## Discussion

This study is the first to randomize methadone initiation in primary care. It was deliberately designed as a pragmatic trial [21], [22], i.e. having a real-life context to ensure external validity and to provide information about methadone effectiveness, irrespective of the induction site. It was also designed to provide recommendations about the possible authorization of methadone induction in primary care in France.

The main result of the trial is that induction in primary care is feasible, as patients in primary care are not less likely to be abstinent from street opioids compared with those inducted in specialized care. Another interesting result is that PC appears to be more attractive for opioid-dependent individuals, first because patients randomized into primary care were more likely to accept treatment than those randomized in specialized centers. Secondly patients who were inducted (after randomization) in primary care were more likely to engage in treatment and report greater satisfaction with medical information provided by their physician. Furthermore, this study highlighted that once methadone treatment was started, retention in treatment was similar in both arms. These last two results reflect the two main criteria generally used for assessing the effectiveness of treatments for opioid dependence - namely retention and non-medical opioid use – and confirm the comparable effectiveness of PC and SC over one year.

Similar results were found in a previous pragmatic trial comparing maintenance in primary care and specialized care. That trial also showed comparable outcomes and better satisfaction with treatment in the primary care maintenance arm [5], [19], [23].

Engagement in treatment was significantly lower in specialized care than in primary care, with early discontinuation rates significantly higher in the former group. This may be directly attributable to the specific French context, where access to buprenorphine already exists in primary care [4]. Indeed, previous pragmatic trials for other treatments involving primary care [15] have highlighted that strong patient engagement is related to the high motivation of physicians enrolled in the trial. This was also the case for physicians who accepted to participate in the present trial. Indeed this is the population of physicians which will be targeted if methadone induction becomes authorized in France.

In addition, it is important to note that the results of this trial by and large are consistent with those found in previous literature [24]. This shows that patients receiving methadone in primary care have comparable treatment responses in terms of retention and opioid use.

Using primary care as an entry point for opioid dependence care (and also for associated comorbidities) has greatly contributed to the scaling-up of treatment for opioid dependence in countries such as the UK. Even more importantly it helps “normalize” care for drug users. The availability of buprenorphine in primary care has partially contributed to “normalize” care for drug users in France as it means they can initiate a treatment option for opioid dependence “free-of-charge” like any other patient, and do so in structures which also provide care for other medical conditions to the general population.

As the French public health authorities were particularly concerned by the overdose risk during induction, the design of the trial proposed a specific model of care for methadone induction in PC in order to maximize safety. Individuals inducted in PC were over-represented in order to better detect possible overdoses during induction in the PC arm. As it happened, no overdoses were observed during induction, confirming previous results about the importance of a shared-care model to minimize such a risk [16].

The strengths of this study lie in the following three key points: first the identification of physicians who will likely be authorized to induct methadone by public health authorities; second the choice of a population of patients which was highly representative of individuals seeking care for opioid dependence in France (non-inclusion criteria were based exclusively on clinical practice criteria to control the risk of overdose); third, the adoption of a flexible treatment protocol where patients could choose to change arm after induction reflecting current clinical practice in France.

One reason why equity of access to methadone and buprenorphine is important in France is because primary care physicians regularly have to manage persistent buprenorphine-injection practices and associated complications [25], which are often a consequence of inadequate dosage prescription [26] or patient dissatisfaction with treatment. For this reason it is important for France to also consider patients who need to switch from buprenorphine to methadone for medical reasons. Despite the wide availability of generic buprenorphine in France, switching from buprenorphine to methadone in primary care could result in reduced costs for care of opioid dependence. However, methadone is likely to present more drug-drug interactions than buprenorphine for those on HIV or HCV medication. Consequently, before starting methadone in patients already receiving such medication, physicians should know how to modify methadone doses accordingly. This is why primary care physicians involved in the present trial received training and appropriate guidelines for all possible drug-drug interactions with methadone treatment, together with guidance about the differences between starting methadone in buprenorphine patients compared with methadone induction in street-opioid users.

A substantial portion of the participants starting methadone in SC switched from buprenorphine to methadone. This is the usual treatment chain for opioid-dependent patients in France. This is not so frequent in other countries. However, switching in France is impossible in areas underserved by specialized centers for substance dependence (i.e. non-urban areas) where general methadone initiation is not available.

Although methadone induction is already possible in primary care in some countries including the UK and Switzerland, no previous trial has compared methadone outcomes over one year as a function of the site of induction. The randomization of a specific model of care for drug users has already been performed in other trials [5], [27], [28] which had similar public health objectives.

The population targeted in this trial is representative of those seeking care for opioid dependence in France [29] and in other countries where similar models of specialized care are available [30]–[33]. However it is difficult to say to what extent these results remain valid in countries where access to opioid maintenance treatment is not free for drug users. Nevertheless, the study was performed according to standard international guidelines [34], [35] in order to make our results as relevant as possible for other contexts.

Among the limitations of the trial, the need for physical proximity between the primary care physician and specialized centers obliged us to target areas where patients' need for methadone was already being substantially met by the specialized centers rather than underserved areas. This resulted in slowed enrolment rates and the enforced extension of the duration of enrolment period. Certainly a larger sample size and longer follow-up would have provided long term comparisons and increased the study's power, but this was not feasible both because of the practical reasons outlined above and cost reasons. The possible effect of heterogeneity between sites (i.e. sites with more than one PC physician versus sites having only one) was tested as a random effect factor in mixed models but was not found to be significant. It is possible that a larger sample size could have altered these results. However, it was important to represent different site sizes (i.e. those with different numbers of participating PC physicians) in the trial for external validity reasons.

Today, many governments of countries with HCV and HIV epidemics driven by drug use are still reluctant to introduce or scale up methadone treatment using alternative models of care, even though such an approach would be cost-effective, especially for controlling HIV and HCV [36].

The model used for primary care in this study may be of interest in other settings where access to methadone is needed for HIV and HCV prevention purposes. However, it is important to remember that our results strongly depend on the specific context of France where primary care already plays an important role in engaging patients in treatment for opioid dependence.

In conclusion, methadone induction in primary care is feasible and acceptable for both physicians and patients. It is as effective as induction in specialized care in reducing street-opioid use and ensuring engagement and retention in treatment for opioid dependence.

## Supporting Information

Checklist S1 **CONSORT 2010 checklist for the ANRS-Methaville trial.**
(DOC)

English protocol S1 **Published protocol of ANRS-Methaville trial.**
(PDF)
