# Supplementary material for: Methadone Induction in Primary Care for Opioid Dependence: A Pragmatic Randomized Trial (ANRS Methaville)
Source: PLoS One. 2014 Nov 13;9(11):e112328. doi: 10.1371/journal.pone.0112328 (PMC4231094; doi:10.1371/journal.pone.0112328)
Supplement: English protocol S1 — Published protocol of ANRS-Methaville trial. (PDF) [file pone.0112328.s002.pdf]

Roux et al. BMC Public Health 2012, 12:488
http://www.biomedcentral.com/1471-2458/12/488

BMC Public Health

STUDY PROTOCOL Open Access

Methadone induction in primary care (ANRS-Methaville): a phase III randomized intervention trial

Perrine Roux[1,2,3*], Laurent Michel[4,5,6], Julien Cohen[1,2,3], Marion Mora[1,2,3], Alain Morel[7], Jean-Francois Aubertin[8], Jean-Claude Desenclos[9], Bruno Spire[1,2,3] Patrizia M Carrieri[1,2,3] and the ANRS Methaville Study Group

Abstract

Background: In France, the rapid scale-up of buprenorphine, an opioid maintenance treatment (OMT), in primary care for drug users has led to an impressive reduction in HIV prevalence among injecting drug users (IDU) but has had no major effect on Hepatitis C incidence. To date, patients willing to start methadone can only do so in a methadone clinic (a medical centre for drug and alcohol dependence (CSAPA) or a hospital setting) and are referred to primary care physicians after dose stabilization. This study aims to assess the effectiveness of methadone in patients who initiated treatment in primary care compared with those who initiated it in a CSAPA, by measuring abstinence from street opioid use after one year of treatment.

Methods/Design: The ANRS-Methaville study is a randomized multicenter non-inferiority control trial comparing methadone induction (lasting approximately 2 weeks) in primary care and in CSAPA. The model of care chosen for methadone induction in primary care was based on study-specific pre-training of all physicians, exclusion criteria and daily supervision of methadone during the initiation phase. Between January 2009 and January 2011, 10 sites each having one CSAPA and several primary care physicians, were identified to recruit patients to be randomized into two groups, one starting methadone in primary care (n = 147), the other in CSAPA (n = 48). The primary outcome of the study is the proportion of participants abstinent from street opioids after 1 year of treatment i.e. non-inferiority of primary care model in terms of the proportion of patients not using street opioids compared with the proportion observed in those starting methadone in a CSAPA.

Discussion: The ANRS-Methaville study is the first in France to use an interventional trial to improve access to OMT for drug users. Once the non-inferiority results become available, the Ministry of Health and agency for the safety of health products may change the the New Drug Application (NDA) of methadone and make methadone induction by trained primary care physicians possible.
The trial is registered with the French Agency of Pharmaceutical Products (AFSSAPS) under the number 2008-A0277-48, the European Union Drug Regulating Authorities Clinical Trials.
Number Eudract 2008-001338-28, the ClinicalTrials.gov Identifier: NCT00657397 and the International Standard Randomised Controlled Trial Number Register ISRCTN31125511.

Keywords: Methadone, Primary care, Initiation, Overdose, Opioid use, Opioid maintenance treatment

* Correspondence: perrine.roux@inserm.fr
[1]INSERM U912 (SESSTIM), Marseille, France
[2]Université Aix Marseille, IRD, UMR-S912, Marseille, France
Full list of author information is available at the end of the article

BioMed Central

© 2012 Roux et al.; licensee BioMed Central Ltd. This is an Open Access article distributed under the terms of the Creative Commons Attribution License (http://creativecommons.org/licenses/by/2.0), which permits unrestricted use, distribution, and reproduction in any medium, provided the original work is properly cited.

Background

Since 1996, two opioid maintenance treatments (OMT) have been marketed in France for opioid dependence: methadone and buprenorphine. It is acknowledged that recommended doses of buprenorphine are less effective than methadone in retaining patients in treatment [1]. Currently, methadone and buprenorphine are accessible through different models of delivery and consequently target different drug-user populations [2]. Buprenorphine is prescribed mainly in primary care, whereas methadone can only be initiated in a methadone clinic (medical centre for drug and alcohol dependence (CSAPA) or in a hospital setting), where patients receive both comprehensive general care and care for psychiatric comorbidities. In addition, in France, a patient can start buprenorphine promptly (even after the first visit) in primary care, whereas methadone induction in a CSAPA can take between 2 and 4 weeks. This is due to the fact that in general the patient must often first complete a psychiatric evaluation and psychosocial and motivational assessments.

Despite the lack of official clinical guidelines about the specific drug(s) to be prescribed in first-line treatment, buprenorphine is prescribed more often and is more widely available in France through primary care. This is due to its safety profile. While the effectiveness of the two treatments at adequate doses (for buprenorphine, this means doses which are higher than those recommended) is comparable, the risk of overdose is lower in buprenorphine patients [1]. As a consequence, buprenorphine is more accessible to individuals who are less severely opioid dependent [3]. Although this model of care for opioid dependence has had a positive impact on HIV epidemics and overdoses in drug users in France, it is still inadequate in terms of reducing the spread of Hepatitis C epidemics (HCV) [4]. Indeed, the persistence of risky behaviours among opioid-dependent drug users could be interpreted as a response to an inadequate model of care. Many do not have access to methadone because of a limited access to CSAPA. In addition, it has been shown that a proportion of buprenorphine patients, albeit marginal, administers the treatment by a non-indicated route, either intranasally [5] or intravenously [6]. Expanded access to methadone treatment for opioid dependent individuals through innovative models of care is a major challenge for the control of HIV and HCV infection. The study described here is based primarily on the identification of a possible model for methadone initiation in primary care. Although the long-term effectiveness of methadone induction in specialized centres on different outcomes, including opioid use, has already been proven [7,8], it is expensive, access is inadequate and many patients perceive it as stigmatizing. For these reasons, we conducted a trial to assess the non-inferiority of the proportion of patients abstinent from street opioid in patients inducted in primary care versus those inducted in CSAPA after one year of methadone treatment.

Methods/Design

Pre-trial phase

The study was designed in response to a request by the French Ministry of Health which asked the AIDS agency (ANRS) to develop an experimental study which would evaluate the effectiveness of methadone in primary care.

The research unit responsible for conducting the study is the unit U912 of the National Institute of Health and Medical Research (INSERM) in Marseilles, under the scientific responsibility of Patrizia Carrieri.

Before designing the trial, a multidisciplinary working group which also included members of patients' associations was set up by the ANRS.

One of the major points to decide upon was the identification of a model for primary care which could maximize access while minimizing the risk of overdose. This model was designed from previous experiences of methadone provision in primary care [9] and particularly the Scottish experience [10]. The main points retained were: 1) study-specific pre-training for primary care physicians; 2) a shared care model, based on the patient-primary care physicians-CSAPA-pharmacist network; 3) the exclusion of patients with triple codependence on opioids/benzodiazepines/alcohol, as screened by MINI; 4) the daily supervision at the local pharmacy during the initiation phase for patients starting methadone in primary care; 5) patient accountability for treatment intake and appropriate storage.

Several subgroups were charged with, respectively, preparing the guidelines for methadone prescription, updating the methadone drug-drug interactions manual and designing an information booklet for the patient.

Participants

From January 2009 to January 2010, we recruited 195 men and women in 10 cities (Avignon, Bayonne, Besancon, Bordeaux, Boulogne, Le Havre, Marseille, Metz, Rouen, Strasbourg) who were all over 18 and less than 70 years old, opioid-dependent in accordance with the DSM-IV criteria, and with an indication for methadone treatment (patients seeking care for opioid dependence and methadone prescription naive for at least one month or patients receiving buprenorphine but needing to switch to methadone treatment for medical reasons). The only difference between this trial and some previous trials [11,12] was that in our study patients who had triple dependence (opioids, benzodiazepines and alcohol) and those who could not be contacted by phone were excluded. The reason for excluding opioid-dependent individuals also presenting with benzodiazepine and alcohol dependence was based on the

results of several studies which have shown an increased risk of overdose associated with benzodiazepine use and alcohol use [13].

Research design

This study is a multi-site, open-label, randomized controlled non-inferiority trial, comparing methadone induction in France in a CSAPA or with primary care physicians with an allocation ratio of 1 :2. The randomization procedure allocated patients to the two different types of prescriber for methadone induction, lasting approximately 14 days for both prescribers).

A pilot phase was first carried out at one site (Avignon CSAPA and local primary care physicians) at a “slowed” recruitment rate in order to identify and prevent possible future problems when the trial was extended to the other sites.

Physician recruitment and training

A one-day training session was provided to all physicians involved in the study to standardize how to initiate methadone treatment (as set down by the trial’s guidelines) and to become acquainted with the design of the trial. This training session also provided the opportunity to collect data about the physicians’ own socio-demographic characteristics and their history of care with opioid-dependent individuals.

We trained 57 prescribers and each site was characterized by a group of trained primary care physicians and a group of trained physicians from a CSAPA. Primary care and CSAPA physicians collaborated closely together to make the randomization process possible and to ensure effective patient follow-up. Concerning patient selection, the Mini-International Neuropsychiatric Interview (MINI) was used to ascertain DSM-IV diagnosis [14] of substance disorders in order to exclude patients with alcohol-benzodiazepines co-dependence. It has been already shown that such patients require closer follow-up [15] and are at greater risk of overdose [13]. During pre-trial training, physicians learned how to use the MINI and familiarized themselves with the trial’s guidelines, including details on the protocol. They also received a manual on drug-drug interactions [16]. One of the main goals of the training session was to remind physicians about the pharmacokinetic profile of methadone during induction i.e. its slow and progressive plasma scale-up before reaching a steady-state. For example the trainer commented that: “An excessive dose given on Monday may lead to an overdose only on Thursday”. The session also helped physicians to identify patients with a high risk of overdose. Furthermore, it emphasized the importance of the information physicians needed to provide patients when initiating methadone. A trial-specific information booklet designed for the patient was drafted explaining the study protocol, all the risks and benefits of initiating methadone, how to deal with complicated or unexpected issues such as travel and holidays, effects on one’s desire to become pregnant, as well as information on intoxication, overdoses (e.g. how to recognize overdose symptoms) and other side effects.

Pharmacist involvement

We also enrolled primary care pharmacists who agreed to deliver methadone and supervise dose taking during the 2-week induction phase (except during weekends, in most cases) for all the trial’s participants. Pharmacists were asked to complete a self-administered questionnaire before and after the trial and also to register all stock entries and exits for methadone doses.

Remuneration

Physicians who accepted to participate in the trial were remunerated for the one-day initial training session and for each medical visit during the induction phase. Participants were covered for all health costs related to their methadone induction.

Intervention

Patient screening

The first visit took place when a patient willing to be treated for his/her opioid dependence came to a Methaville physician, either in primary care or in a CSAPA (see Figure 1). During this visit, the physician explained the study protocol to the patient and performed the trial screening to confirm the patient’s eligibility. If he/she was eligible to enter the study and provided written informed consent to participate, the physician then immediately called the centre of methodology and management (CMM) of unit U912 to randomize the patient into one of the two groups. Details were exchanged and at the end of the medical visit the participant knew where he/she had to initiate methadone: in primary care or in a CSAPA.

This study was approved by the Ethics Committee of Persons Protection in Paris, France.

The screening physician, whether in a CSAPA or in a primary care setting, provided the randomization setting to the patient: if the patient was randomized into the same type of setting at that where initial screening took place, he/she could initiate methadone where he/she was screened. Otherwise he/she had to go to the other type of setting.

Methadone initiation

During the induction period, the selected physician for the methadone induction had to adjust the methadone dose until stabilization was reached. This induction period was crucial and required a great deal of attention by both the physician and the patient in order to reach a

balance between overdose risk reduction and withdrawal symptoms' alleviation. The initial methadone prescription could not be above 40 mg a day. After writing this first prescription, the physician was required to call the local pharmacist where the patient had chosen to take the methadone. The patient was then given the prescribed dose (in syrup form) at the pharmacy and had a medical visit 2 or 4 hours after that intake in order to assess the tolerance of the first prescribed dose. During the induction period, the patient had to see the physician at least twice a week after the initial prescription consultation. Depending on the severity of opioid dependence, the first dose prescribed could increase by 10 mg every 2 to 4 days with a medical visit 48 hours after each dose increase.

Maintenance phase

After the initial 14 days, the participant could choose to remain in the allocated prescription setting or to change to the other one (i.e. to primary care or CSAPA).

The possible risks for participants were methadone side effects, which are the same as those encountered in standard clinical care: drowsiness, sweating, constipation, sleeping and libido disorder [17]. The potential benefit for patients initiating in primary care was that they would have the chance to initiate methadone in primary care, something not currently possible in the standard system of care.

As there is an increased risk of overdose until stabilization of doses is reached, special guidelines for methadone prescription were drawn up and used, both to train physicians and to have standardized guidelines throughout the course of the trial.

Lost to follow-up

During the induction phase (from day 0 to day 14), the patient had to:

(1) regularly go to the pharmacy (according to the scheduled date of prescription refill) during opening hours to take the treatment. If he/she didn't appear there for more than 48 hours, the pharmacist had to inform the centre of methodology and management (CMM) which in turn contacted the participant.
(2) see the physician for the scheduled medical visit, every 72 hours on average. If this appointment was not observed, the physician had to call the CMM.

If after 24 hours of investigation, the CMM still could not find the participant in either of these two cases, he/she was considered as "lost to follow-up". The CMM had to then contact the centre of evaluation and information on drug dependence (CEIP) to check that the participant had not had an overdose.

Safety

An independent data and safety monitoring board periodically reviewed the trial's efficacy and safety data. This safety committee comprised several experts in the field including addiction psychiatrists, forensic scientists, pharmacologists, methodologists and institutional scientists.

Stopping rules were based on occurrence of overdoses and severe adverse effects.

Objectives

The trial is nearing completion. Its primary objective is to demonstrate the non-inferiority of the proportion of individuals abstinent from street opioids after one year of methadone in patients inducted in primary care with those inducted in CSAPA. Patients who initiated methadone in primary care are expected to have better outcomes compared with those in CSAPA in terms of retention, quality of life and satisfaction after 1 year of methadone treatment.

Outcomes and instruments

Multiple sources were used for data collection at screening (week preceding recruitment and first/seventh day after recruitment), at recruitment (day zero or month zero), induction (from the first to the second week of treatment) and maintenance (third, sixth and twelfth months). Data collection methods (ongoing) include face-to-face interviews, phone interviews using a Computer Assisted Telephone Interview [18] and self-administered questionnaires, all adapted and validated for the type of information to be gathered.

The Opiate Treatment Index (OTI), a multi-dimensional questionnaire based on patients' self-report [19] is being used to measure Methaville's primary outcome. This consists in measuring the proportion of all participants abstinent from street opioids after 1 year of treatment. Some secondary outcomes are also measured. First, retention in treatment and occurrence of overdoses are assessed. The prevalence of other HCV risk transmission practices is also documented using a series of questions extracted from a standardized questionnaire specifically adapted for this purpose - the Blood-Borne Virus Transmission Risk Assessment (BBV-TRAQ) [20,21] - and also the questionnaire used by Lucidarme et al. in their longitudinal study [22,23]. Other data about indicators relevant for the purpose of this study have been collected: depressive symptoms (using the CES-D questionnaire [24]), suicidal risk (the Beck Hopelessness Scale [25]), impulsivity (the Barratt Impulsiveness Scale), sensation seeking (the Brief Sensation Seeking Scale (BSSS) [26]), tobacco dependence (the Fagerstrom test [27]), alcohol consumption (the AUDIT questionnaire [28]), pain assessment (the Brief Pain Inventory [29]), adherence to methadone prescription, patient-

health care provider relationship [30], opioid withdrawal (the subjective opioid withdrawal symptoms scale (SOWS) [31]), quality of life (the 12-item Short-Form Health Survey (SF-12) [32]), Attention Deficit Hyperactivity Disorder (Adult ADHD Self-Report Scale 6 item version [33]), urinary drug screening, and finally socio-demographic information on history of incarceration and contact with associations. Quality assurance and data security measures were established and approved for this protocol on the 21st of May 2008.

Sample size

We used the proportion of all patients abstinent from street opioids after 1-year of methadone treatment for the sample size. Many studies have shown that 60% to 80% of opioid-dependent individuals treated with methadone completely stopped using street opioids after one year of treatment [34-36]. The hypothesis for this study was that after one year of methadone treatment, the proportion of patients not using opioids in CSAPA would be 70%. Considering a margin of inferiority of 15% in the primary care arm, 200 patients needed to be recruited to investigate non-inferiority.

Randomization

For allocation of the participants to a primary care or a CSAPA physician, a computer-generated list of random numbers was used within a secured centralized internet system. Randomization sequencing was not stratified by the CMM, which generated the randomization sequence by computer and provided the number by phone to the prescribing physicians when recruiting a patient into the study.

Blinding

By definition, the arm could not be masked from the prescribing physician but it was masked from all the scientific team involved in the trial. It was obviously unmasked to the logistics team who conducted the interviews and to statisticians and the data managers of the research group, because they had to regularly meet and submit reports to the Independent Committee of the trial. The study will be unmasked at the end of the last M12 interview, during the course of 2012.

Statistical methods

The trial was initially designed as a non-inferiority trial aimed to show the non-inferiority of the proportion of *non-injectors* who started methadone in primary care compared to those who started it in a CSAPA. However, as the number of patients injecting opioids at recruitment was low, the primary outcome was changed as 'the proportion of *patients abstinent from* of street opioids after 1 year', and the dimension of the study was re-computed. These changes were approved by the Scientific Committee of the trial, the Independent Committee and the Ethical Committee (CPP).

Secondary outcomes are the occurrence of fatal and non-fatal overdoses and the percentage of retention in both arms. We also investigated the prevalence of other HCV transmission practices and the effectiveness of treatment in terms of adherence, social insertion, addictive behaviours, quality of life, psychiatric comorbidities, social insertion, reduction in criminal acts and cost effectiveness.

When data analysis is completed later in 2012, the computation of the 95% confidence interval for the proportion of patients abstinent from street opioids at M12 in the two arms will be based on Intention To Treat (ITT), with lost to follow-up considered as a therapeutic failure (opioid users). Additional analyses will be performed without ITT, focusing on the most recent available information about opioid use during treatment. Mixed generalized linear models analyses will be used to identify predictors of the different outcomes. Non-inclusion or non-termination biases will be controlled for using selection models - including Heckman type models [37] - and other approaches to take into account for the drop out process [38].

Discussion

To date, the ANRS-Methaville study is the first in France to be requested by a governmental agency with the objective of potentially changing the existing legal framework of an opioid maintenance treatment (OMT). We hope that methadone will be initiated by trained primary care physicians and in turn, that it will contribute to providing improved access to methadone in France.

However, the risk of overdose occurring during the induction phase of treatment [39] highlights the requirement for closer follow-up early on, together with the systematic training of volunteer physicians. For many years now, in the United Kingdom, methadone has been mostly prescribed in primary care (as opposed to special centres) [9]. This system of access to methadone has shown its effectiveness but also the risks associated with widespread access [40,41]. In Scotland, methadone dose supervision in community pharmacies is currently in place [10,42].

This is why the present trial is based not only on guidelines for methadone prescription and provision in primary care but also on a shared-care model.

Primary care physicians should work within a drug dependence care network and/or specialized centre, such as a CSAPA, and have documented experience of care for drug users. In addition, pharmacists must have direct contact with the methadone prescribing physician and must be involved from the beginning of the trial in order to guarantee effective supervision and follow-up of

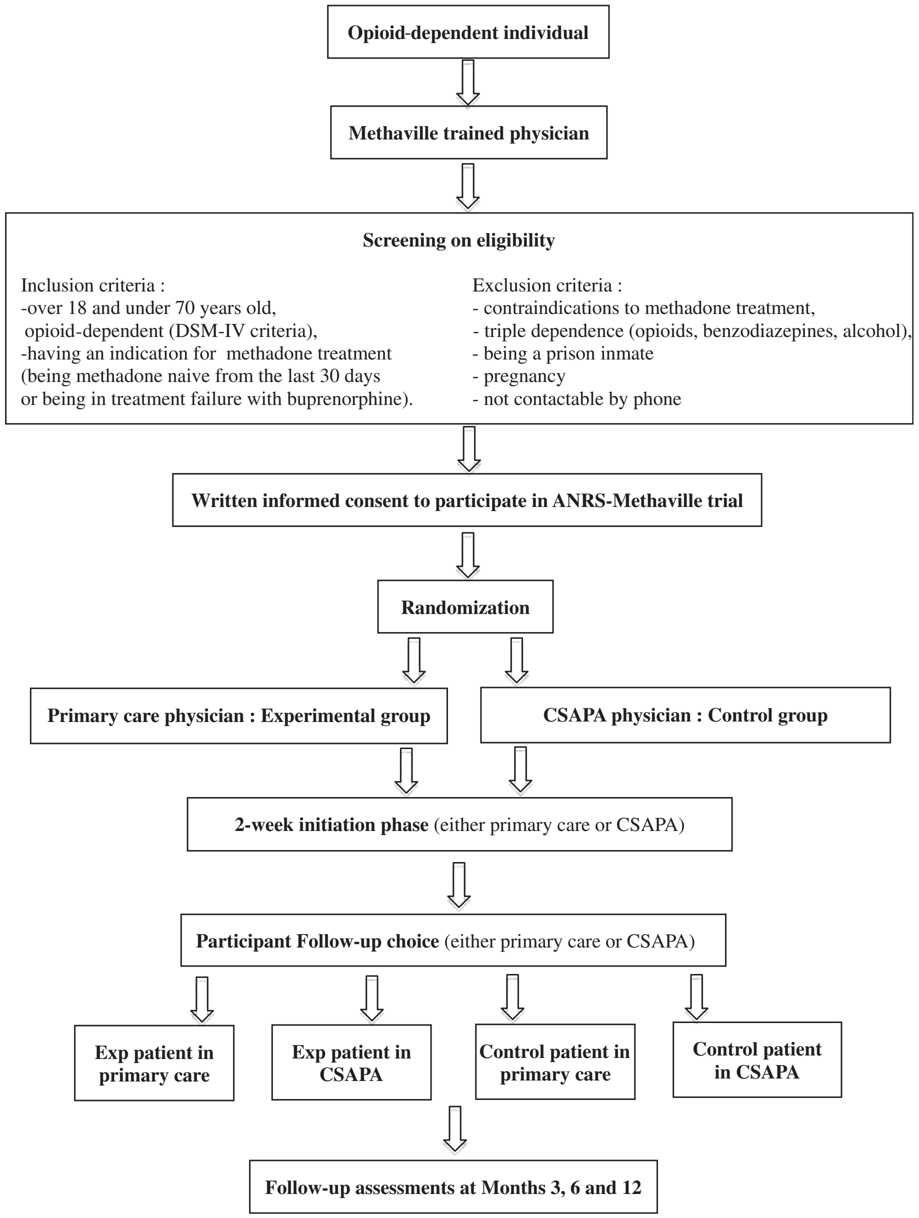

Figure 1 Flowchart for patient inclusion and study design.

patients. This important responsibility may integrate pharmacists more into the health care system for drug users. Some previous studies have already shown that pharmacists appear to be in favour of providing OMT, supervising intake [43] and playing an important role in treatment delivery, including referring difficult patients to other services [44]. This potentially enhanced role for pharmacists is a unique opportunity to develop and improve the harm reduction network through the training of pharmacists.

This study may inform the development of any improved structuring of the existing framework for OMT induction in primary care through training and networking between physicians and pharmacists. The improved treatment efficacy of involving pharmacists as a key component of prevention for drug users has

already been shown through the widespread provision of sterile injecting equipment [45].

In this trial, because the recruitment of primary care physicians had to be carried out in close collaboration with a drug and alcohol centre, it was impossible to include primary care physicians in sites without any CSAPA (especially in rural settings where primary care physicians are mostly general practitioners). The representativeness of the study sample would certainly have been better if we had been able to include such rural-based physicians. However, we can hypothesize that whatever results we find will also be applicable to these latter as our intervention included pre-training for all the physicians who initiated methadone treatment.

This trial is a unique opportunity to encourage increased access to OMT, especially for opioid dependent individuals who do not have access to methadone for geographical reasons or for reasons of preference. In addition, it is an innovative experiment in France using preventive intervention research in opioid dependent individuals.

Abbreviations

AFSSAPS: French Agency of Pharmaceutical Products; ANRS: Aids Agency; CEIP: Centre of evaluation and information on drug dependence; CMM: Centre of methodology and management; CPP: Committee and the Ethical Committee; CSAPA: Medical centre for drug and alcohol dependence; HCV: Hepatitis C virus; HIV: Human immunodeficiency virus; IDU: Injecting drug user; INSERM: National Institute of Health and Medical Research; ITT: Intention to treat; MINI: Mini-International Neuropsychiatric Interview; OMT: Opioid Maintenance Treatment; OTI: Opiate Treatment Index.

Competing interests

All authors have no financial interests that may be relevant to the submitted work.

Authors' contributions

MPC, LM, PR, BS, JFA, AM and JCD designed the study and wrote the protocol. PR and MPC managed the literature searches, formulated the research questions and PR wrote the first draft of the manuscript. JC and MM participated in the data collection and JC will undertake the statistical analyses. All authors contributed to and have approved the final manuscript.

Acknowledgements

The study received external funding by the French National Agency for Research on Aids and Viral Hepatitis (ANRS) and the French Ministry of Health. We thank all members of the ANRS-Methaville Study Group. We especially thank all physicians involved in the trial and all patients who took part in this study. Finally, we thank Jude Sweeney for the English revision and editing of our manuscript.
The ANRS Methaville Study Group
Scientific Committee: P.M. Carrieri (principal investigator), A. Morel (principal investigator), L. Michel, M. Mora, P. Roux, JF. Aubertin, S. Robinet, JC. Desenclos, J. Cohen, A. Herszkowicz, C. Paul, I. Porteret, T. Sainte Marie.
Safety committee: J. Bachellier, P. Beauverie, JP Couteron, C. Marty-Double, M. Vray.
International committee of experts: R. Baker, H. Catania, M. Gossop, R. Haemmig, M. Torrens, A. Wodak.
Physicians: Dr Achard; Dr Aizpurua; Dr Aubertin; Dr Azalbert; Dr Balteaubijeau; Dr Bartolo; Dr Berger; Dr Bibette; Dr Biderman; Dr Blochlaine; Dr Cadart; Dr Casenave; Dr Castera; Dr Causse; Dr Corlieu; Dr Delile; Dr Demasse; Dr Depieds; Dr Dewost; Dr Drosson; Dr Duffez; Dr Faust; Dr Gassmann; Dr Guena; Dr Guillet; Dr Gutenkust; Dr Herouin; Dr Herran; Dr Heitz; Dr Kerloc'h; Dr Khouri; Dr Lang; Dr Lasalarie; Dr Lavignasse; Dr Magnin; Dr Maire; Dr Margelis; Dr Marre; Dr Mauraycaplanne; Dr Michel; Dr Morel; Dr Nemayech; Dr Noss; Dr Pages; Dr Paillou; Dr Partouche; Dr Petit; Dr Pouclet; Dr Raulin; Dr Regard; Dr Reiller; Dr Roch; Dr Rouille; Dr Sananes; Dr Sangare; Dr Truffy; Dr Vergez; Dr Vincent; Dr Wajsbrot.
Centre of Methodology and Management, INSERM U912:
Protocol, guidelines, training: MP. Carrieri L. Michel, M. Mora, P. Roux.
Phone interviews, training, logistics: G. Maradan, J. Biemar, S. Huguet, C.Bravard.
Data collection, management and statistical analyses: P. Kurkdji, C. Taieb, J. Cohen, C.Lions.
Administration: C. Giovannini, MP. Kissikian.
AIDS agency (ANRS): JC. Desenclos, N. Job-Spira, V. Dore, C. Paul, I. Porteret.
French agency for the safety of health products (AFSSPAS): N. Richard.
French Ministry of Health: A. Herszkowicz, N. Prisse.
ASUD association: F. Olivet; *AIDES association Centre of evaluation and information on drug dependence (CEIP): J.Arditti.*

Author details

[1]INSERM U912 (SESSTIM), Marseille, France. [2]Université Aix Marseille, IRD, UMR-S912, Marseille, France. [3]ORS PACA, Observatoire Régional de la Santé Provence Alpes Côte d'Azur, Marseille, France. [4]INSERM, Research Unit 669, Paris, France. [5]Univ Paris-Sud and Univ Paris Descartes, UMR-S0669, Paris, France. [6]Centre Pierre Nicole, Paris, France. [7]Oppelia, Paris, France. [8]Thionville, France. [9]Institut de Veille Sanitaire, Saint-Maurice, France.

Received: 14 March 2012 Accepted: 3 April 2012
Published: 28 June 2012

References

1. Mattick RP, Kimber J, Breen C, Davoli M: **Buprenorphine maintenance versus placebo or methadone maintenance for opioid dependence.** *Cochrane Database Syst Rev* 2008, (2):CD002207.
2. Michel L, Roux P, Cohen J, Carrieri MP: **A French prospective observational study of the treatment of chronic hepatitis C in drug abusers: a commentary.** *Gastroenterol Clin Biol* 2009, 33(1 Pt 1):15–16. author reply 16–17.
3. Carrieri MP, Amass L, Lucas GM, Vlahov D, Wodak A, Woody GE: **Buprenorphine use: the international experience.** *Clin Infect Dis* 2006, **43**(Suppl 4):S197–S215.
4. Emmanuelli J, Desenclos JC: **Harm reduction interventions, behaviours and associated health outcomes in France, 1996–2003.** *Addiction* 2005, **100**(11):1690–1700.
5. Roux P, Villes V, Bry D, Spire B, Feroni I, Marcellin F, Carrieri MP: **Buprenorphine sniffing as a response to inadequate care in substituted patients: results from the Subazur survey in south-eastern France.** *Addict Behav* 2008, **33**(12):1625–1629.
6. Vidal-Trecan G, Varescon I, Nabet N, Boissonnas A: **Intravenous use of prescribed sublingual buprenorphine tablets by drug users receiving maintenance therapy in France.** *Drug Alcohol Depend* 2003, **69**(2):175–181.
7. Gossop M, Marsden J, Stewart D, Kidd T: **The National Treatment Outcome Research Study (NTORS): 4–5 year follow-up results.** *Addiction* 2003, **98**(3):291–303.
8. Hubbard RL, Craddock SG, Anderson J: **Overview of 5-year followup outcomes in the drug abuse treatment outcome studies (DATOS).** *J Subst Abuse Treat* 2003, **25**(3):125–134.
9. Gossop M, Marsden J, Stewart D, Lehmann P, Strang J: **Methadone treatment practices and outcome for opiate addicts treated in drug clinics and in general practice: results from the National Treatment Outcome Research Study.** *Br J Gen Pract* 1999, **49**(438):31–34.
10. Weinrich M, Stuart M: **Provision of methadone treatment in primary care medical practices: review of the Scottish experience and implications for US policy.** *JAMA* 2000, **283**(10):1343–1348.
11. Fiellin DA, O'Connor PG, Chawarski M, Pakes JP, Pantalon MV, Schottenfeld RS: **Methadone maintenance in primary care: a randomized controlled trial.** *JAMA* 2001, **286**(14):1724–1731.
12. Strain EC, Bigelow GE, Liebson IA, Stitzer ML: **Moderate- vs high-dose methadone in the treatment of opioid dependence: a randomized trial.** *JAMA* 1999, **281**(11):1000–1005.
13. Kerr T, Fairbairn N, Tyndall M, Marsh D, Li K, Montaner J, Wood E: **Predictors of non-fatal overdose among a cohort of polysubstance-using injection drug users.** *Drug Alcohol Depend* 2007, **87**(1):39–45.
14. Sheehan DV, Lecrubier Y, Sheehan KH, Amorim P, Janavs J, Weiller E, Hergueta T, Baker R, Dunbar GC: **The Mini-International Neuropsychiatric Interview (M.I.N.I.): the development and validation of a structured**

diagnostic psychiatric interview for DSM-IV and ICD-10. *J Clin Psychiatry* 1998, **59**(Suppl 20):22–33. quiz 34–57.
15. Brands B, Blake J, Marsh DC, Sproule B, Jeyapalan R, Li S: **The impact of benzodiazepine use on methadone maintenance treatment outcomes.** *J Addict Dis* 2008, **27**(3):37–48.
16. Leavitt SB: *Methadone-Drug Interactions*. Forum: In Addiction Treatment; 2005.
17. Trafton JA, Ramani A: **Methadone: a new old drug with promises and pitfalls.** *Curr Pain Headache Rep* 2009, **13**(1):24–30.
18. Groves RM, Mathiowetz NA: **Chapter V. A comparison of CATI and non-CATI questionnaires.** *Vital and health statistics* 1987, (106):33–39.
19. Darke S, Hall W, Wodak A, Heather N, Ward J: **Development and validation of a multi-dimensional instrument for assessing outcome of treatment among opiate users: the Opiate Treatment Index.** *Br J Addict* 1992, **87**(5):733–742.
20. Fry CL, Lintzeris N: **Psychometric properties of the Blood-borne Virus Transmission Risk Assessment Questionnaire (BBV-TRAQ).** *Addiction* 2003, **98**(2):171–178.
21. Stoove MA, Fry CL, Lintzeris N: **Quantifying hepatitis C transmission risk using a new weighted scoring system for the Blood-Borne Virus Transmission Risk Assessment Questionnaire (BBV-TRAQ): applications for community-based HCV surveillance, education and prevention.** *Harm Reduct J* 2008, **5**:12.
22. Lucidarme D, Foutrein P, Creusy C, Forzy G, Foutrein-Comes MC, Muyssen A, Bailly D, Parquet PJ, Filoche B: **Prevalence of hepatitis C, B and D markers and histopathological aspects in a group of intravenous drug addicts.** *Gastroenterol Clin Biol* 1994, **18**(11):964–968.
23. Lucidarme D, Bruandet A, Ilef D, Harbonnier J, Jacob C, Decoster A, Delamare C, Cyran C, Van Hoenacker AF, Fremaux D, *et al*: **Incidence and risk factors of HCV and HIV infections in a cohort of intravenous drug users in the North and East of France.** *Epidemiol Infect* 2004, **132**(4):699–708.
24. Fuhrer R, Rouillon F: **La version française de l'échelle CES-D. Description and translation of the autoevaluation scale (in French).** *Psychiatrie et Psychobiologie* 1989, **4**:163–166.
25. Beck AT, Steer RA, Kovacs M, Garrison B: **Hopelessness and eventual suicide: a 10-year prospective study of patients hospitalized with suicidal ideation.** *Am J Psychiatry* 1985, **142**(5):559–563.
26. Roberti JW, Storch EA, Bravata E: **Further psychometric support for the Sensation Seeking Scale–Form V.** *J Pers Assess* 2003, **81**(3):291–292.
27. Etter JF, Duc TV, Perneger TV: **Validity of the Fagerstrom test for nicotine dependence and of the Heaviness of Smoking Index among relatively light smokers.** *Addiction* 1999, **94**(2):269–281.
28. Saunders JB, Aasland OG, Babor TF, de la Fuente JR, Grant M: **Development of the Alcohol Use Disorders Identification Test (AUDIT): WHO Collaborative Project on Early Detection of Persons with Harmful Alcohol Consumption–II.** *Addiction* 1993, **88**(6):791–804.
29. Poundja J, Fikretoglu D, Guay S, Brunet A: **Validation of the French version of the brief pain inventory in Canadian veterans suffering from traumatic stress.** *J Pain Symptom Manage* 2007, **33**(6):720–726.
30. Smith MY, Winkel G, Egert J, Diaz-Wionczek M, DuHamel KN: **Patient-physician communication in the context of persistent pain: validation of a modified version of the patients' Perceived Involvement in Care Scale.** *J Pain Symptom Manage* 2006, **32**(1):71–81.
31. Gossop M: **The development of a Short Opiate Withdrawal Scale (SOWS).** *Addict Behav* 1990, **15**(5):487–490.
32. Ware J Jr, Kosinski M, Keller SD: **A 12-Item Short-Form Health Survey: construction of scales and preliminary tests of reliability and validity.** *Med Care* 1996, **34**(3):220–233.
33. Kessler RC, Adler L, Ames M, Demler O, Faraone S, Hiripi E, Howes MJ, Jin R, Secnik K, Spencer T, *et al*: **The World Health Organization Adult ADHD Self-Report Scale (ASRS): a short screening scale for use in the general population.** *Psychol Med* 2005, **35**(2):245–256.
34. Smyth BP, Barry J, Lane A, Cotter M, O'Neill M, Quinn C, Keenan E: **In-patient treatment of opiate dependence: medium-term follow-up outcomes.** *Br J Psychiatry* 2005, **187**:360–365.
35. Maremmani I, Pani PP, Pacini M, Perugi G: **Substance use and quality of life over 12 months among buprenorphine maintenance-treated and methadone maintenance-treated heroin-addicted patients.** *J Subst Abuse Treat* 2007, **33**(1):91–98.
36. Wittchen HU, Apelt SM, Soyka M, Gastpar M, Backmund M, Golz J, Kraus MR, Tretter F, Schafer M, Siegert J, *et al*: **Feasibility and outcome of substitution treatment of heroin-dependent patients in specialized substitution centers and primary care facilities in Germany: a naturalistic study in 2694 patients.** *Drug Alcohol Depend* 2008, **95**(3):245–257.
37. Carrieri MP, Leport C, Protopopescu C, Cassuto JP, Bouvet E, Peyramond D, Raffi F, Moatti JP, Chene G, Spire B: **Factors associated with nonadherence to highly active antiretroviral therapy: a 5-year follow-up analysis with correction for the bias induced by missing data in the treatment maintenance phase.** *J Acquir Immune Defic Syndr* 2006, **41**(4):477–485.
38. Protopopescu C, Marcellin F, Spire B, Preau M, Verdon R, Peyramond D, Raffi F, Chene G, Leport C, Carrieri MP: **Health-related quality of life in HIV-1-infected patients on HAART: a five-years longitudinal analysis accounting for dropout in the APROCO-COPILOTE cohort (ANRS CO-8).** *Qual Life Res* 2007, **16**(4):577–591.
39. Caplehorn JR, Drummer OH: **Mortality associated with New South Wales methadone programs in 1994: lives lost and saved.** *Med J Aust* 1999, **170**(3):104–109.
40. Cornish R, Macleod J, Strang J, Vickerman P, Hickman M: **Risk of death during and after opiate substitution treatment in primary care: prospective observational study in UK General Practice Research Database.** *BMJ* 2010, **341**:c5475.
41. Morgan OW, Johnson H, Rooney C, Seagroatt V, Griffiths C: **Changes to the daily pattern of methadone-related deaths in England and Wales, 1993–2003.** *J Public Health (Oxf)* 2006, **28**(4):318–323.
42. Greenwood J: **Creating a new drug service in Edinburgh.** *BMJ* 1990, **300**(6724):587–589.
43. Lawrinson P, Roche A, Terao H, Le PP: **Dispensing opioid substitution treatment: practices, attitudes and intentions of community-based pharmacists.** *Drug Alcohol Rev* 2008, **27**(1):47–53.
44. Sheridan J, Manning V, Ridge G, Mayet S, Strang J: **Community pharmacies and the provision of opioid substitution services for drug misusers: changes in activity and attitudes of community pharmacists across England 1995–2005.** *Addiction* 2007, **102**(11):1824–1830.
45. Bonnet N: **Pharmacy syringe exchange program for injection drug users.** *Presse Med* 2006, **35**(12 Pt 1):1811–1818.

doi:10.1186/1471-2458-12-488
Cite this article as: Roux *et al.*: Methadone induction in primary care (ANRS-Methaville): a phase III randomized intervention trial. *BMC Public Health* 2012 **12**:488.

Submit your next manuscript to BioMed Central and take full advantage of:

- **Convenient online submission**
- **Thorough peer review**
- **No space constraints or color figure charges**
- **Immediate publication on acceptance**
- **Inclusion in PubMed, CAS, Scopus and Google Scholar**
- **Research which is freely available for redistribution**

Submit your manuscript at
www.biomedcentral.com/submit

BioMed Central
